# Actor–Partner Interdependence Between Parenting Perfectionism and Parental Stress Among Chinese Parents Over Time

**DOI:** 10.1111/famp.70146

**Published:** 2026-04-14

**Authors:** Janet T. Y. Leung, Ann W. S. Hung, Xiangying Ding, Vincent W. T. Chua

**Affiliations:** ^1^ Department of Applied Social Sciences The Hong Kong Polytechnic University Hong Kong China

**Keywords:** actor–partner interdependence modeling, Chinese, marital satisfaction, parental stress, parenthood, parenting perfectionism

## Abstract

In contemporary society, parents have become obsessive on achieving “perfection” in preparing their children to succeed in the competitive environment. However, the personal and inter‐spousal relationships between parenting perfectionism and parental stress among Chinese couples over time have been largely overlooked in the academic literature. Additionally, the moderating effect of marital satisfaction on these relationships remains unclear. Based on a sample of 642 Chinese couples with adolescent children in Grades 7 and 8 at Time 1, Actor–Partner Interdependent Modeling (APIM) analyses were conducted to assess the patterns of father–mother interdependence in the associations of parenting perfectionism (adaptive and maladaptive) with parental stress over time, as well as the moderating role of marital satisfaction in the relationships. The findings indicated that mother‐reported maladaptive parenting perfectionism was positively associated with both paternal and maternal stress over time. An actor‐only pattern was identified in the association of maladaptive parenting perfectionism with maternal stress, whereas a partner‐only pattern was observed in the relationship between maladaptive parenting perfectionism and paternal stress. Moreover, father‐perceived marital satisfaction strengthened these associations. These results offer valuable insights for family scholars and practitioners, underscoring the importance of examining the effects of parenting perfectionism on parental well‐being. This understanding is crucial for exploring the dynamics of parenthood in the modern era.

## 1 Introduction

Parenthood is a cherishing developmental stage within the family life cycle (Nomaguchi and Milkie 2020). However, the rise of global meritocracy as well as excessive competition in education in Hong Kong and China in recent decades have driven Chinese parents to strive for “perfection” in preparing their children to succeed in the competitive “rug rat race” (Leung 2022). Parents often fear that any mistakes or inadequacies in parenting can jeopardize their children's future, prompting them to excel in child socialization. Douglas and Michaels (2004) coined the term “New Momism” to describe a form of motherhood where mothers adopt exceptionally high ideals and norms that are difficult to be achieved. In Asian countries, children's achievements have become a “report card” reflecting parental performance and success (Ng et al. 2014).

While majority of related research views perfectionism as an individual personality trait of parents (i.e., a perfectionistic parent; Soenens et al. 2005), studies examining parenting perfectionism as a manifestation of parental roles (i.e., an individual's tendency to be a “perfect parent”) are scarce. Even parents who are not inherently perfectionists may strive to be “perfect parents” by providing the best resources for their children to achieve success. Indeed, it is important to examine parenting perfectionism and parental stress among parents of adolescent children. On the one hand, parents are required to grant greater autonomy to their children, enabling them to explore their self‐identity and develop their competence (Ryan and Deci 2000). On the other hand, parents often harbor concerns regarding their children's ability to navigate the tumultuous transition of adolescence (Arnett 1999). This concern is particularly pronounced among parents who demonstrate high levels of parenting perfectionism, as they tend to be sensitive to mistakes and imperfections that could impact their children's future (Leung 2022).

Moreover, according to family systems theory (Cox and Paley 2003), fathers and mothers are integral parts of the family and they are interdependent, particularly in Chinese families where relationships among members are highly emphasized (Chao and Tseng 2002). However, while many studies have focused solely on the individual effect of perfectionism on one's well‐being (Song et al. 2023), the interspousal associations between parenting perfectionism and parental well‐being are largely ignored in the family literature. Furthermore, quality of marital relationship is a critical factor that may influence the interspousal relationships between parenting perfectionism and parental stress (Hess 2008), yet research on the moderating role of marital relationship remains almost non‐existent.

Methodologically, many studies employing the Actor–Partner Interdependence Model (APIM; Kenny et al. 2006) reported the statistically significance of actor and partner effects to represent patterns of dyadic interdependence (Brenning et al. 2024). Kenny and Ledermann (2010) argued that simply assessing the statistical significance of actor and partner effects may not adequately determine patterns of dyadic interdependence. They therefore recommended the use of a parameter—the ratio of the partner effect to the actor effect (*k*)—to examine different patterns (actor‐only, partner‐only, couple, and contrast) of dyadic interdependence (see Section 2). Recently, an increasing number of family researchers have adopted this approach to examine patterns of couple interdependence in family processes (Jiang et al. 2020).

Finally, many related studies employ a cross‐sectional research design (Lee et al. 2012), which limits the ability to establish cause‐and‐effect relationships. Therefore, this study examined patterns of father–mother interdependence in the associations between parenting perfectionism and parental stress among Chinese parents with adolescent children over time. Additionally, the moderating roles of father‐ and mother‐perceived marital satisfaction (MS) in these associations were assessed.

### 1.1 Parenting Perfectionism and Parental Stress

Parenting perfectionism originates from the concept of personal perfectionism as applied to the realm of parenting (Snell Jr et al. 2005). Perfectionism is defined as an individual's adherence to exceptionally high personal standards and a strong desire to perform flawlessly (Frost et al. 1990). Parenting perfectionism specifically refers to a parent's exceptionally high standards for their own parenting (Snell Jr et al. 2005). Leung (2022) identified four dimensions of parenting perfectionism, subsumed under two hierarchical orders: adaptive parenting perfectionism (APP), which refers to parental orderliness in performing parenting duties, and maladaptive parenting perfectionism (MPP), which encompasses exceptionally high standards in parenting practice, over‐sensitivity to parenting mistakes, and doubts about parenting decisions and actions. While MPP was positively related to parents' depression and negatively associated with their life satisfaction, APP was negatively associated with depression and positively linked with life satisfaction among fathers (Leung 2022).

Parental stress refers to subjective feelings of pressures experienced by parents in fulfilling child‐rearing roles (Cheung 2000). Role identity theory posits that one's identity is constructed upon role expectations embedded within social relationships (Stryker 1987). Personal standards regarding parenting establish a benchmark for what constitutes a “good parent,” thereby enabling individuals to develop a “parent identity” by endorsing and internalizing a set of role expectations associated with this benchmark (Cast 2004). When parents effectively fulfill their expected parental roles, they cultivate a sense of self‐control and a positive “parent identity,” which is advantageous to their overall well‐being (Stoeber and Otto 2006). Conversely, if parents perceive themselves as failing to meet these expectations, they may experience parenting role strain and feel incapable of being “good parents” (Nomaguchi and Milkie 2020), potentially resulting in stress, shame, or even anxiety and depression (Cast 2004; Liss et al. 2013). As APP represents parent's capacity to enforce parenting rules and practice, which fulfill their ideal parental role, thereby enhances their psychological well‐being and reduces parental stress (Stoeber and Otto 2006). In contrast, MPP denotes parental doubt regarding their parenting capacity and decisions (Leung 2022), which may impede their positive parental role identity and consequently contribute to parental stress and anxiety (Cast 2004).

In Chinese culture where familism and collectivism are emphasized, parents feel obligated to raise their children to achieve excellence, thereby bringing honor to the family name (Chao and Tseng 2002). As parenting performance is closely linked to family pride, Chinese parents often set high parenting standards and fear that any flaws or imperfections in their parenting may jeopardize their children's future, leading to increased stress and anxiety (Leung 2022). An empirical study conducted among Chinese parents found that MPP was positively associated with parental stress (Leung 2022). Interestingly, the findings revealed that father‐perceived APP was negatively related to paternal stress, whereas mother‐perceived APP was positively related to maternal stress (Leung 2022). As fathers are typically goal‐oriented and instrumental in parenting practice (Power and Shanks 1989), APP reflects paternal orderliness in establishing rules and guidelines for their children, which leads to a clearer paternal role identity and, consequently, reduces stress. On the contrary, mothers are generally more caring and responsive to their children (Yaffe 2023). APP represents mother's meticulousness in performing caregiving duties (Leung 2022), which may create higher stress.

### 1.2 Interdependence Across Parent Gender

According to family systems theory (Cox and Paley 2003), fathers and mothers are integral components of the family system and are interconnected, suggesting that one's parenting perfectionism may also be linked to the spouse's parental stress. Couples often expect a high degree of concordance in attitudes and approaches when socializing their children (Deschênes et al. 2014). When a parent exhibits high parenting perfectionism, one may demand the spouse to adhere to similar standards, which may create stress for the spouse. In fact, Brenning et al. (2024) identified that a mother‐reported MPP was positively linked to both her own and her spouse's burnout, whereas a father‐reported MPP was positively associated with his own burnout. Although fathers are increasingly involved in parenting roles, mothers still bear a greater share of parenting responsibilities (Nomaguchi and Milkie 2020). When confronted with extremely high standards set by the spouse, fathers may feel inadequate and fatigued in parenting (Brenning et al. 2024). However, studies examining the crossover effects of APP on parental stress are currently lacking. When one parent is particularly strict regarding parenting orderliness, he or she may expect the spouse to adopt a similar approach; failure to do so may create marital conflict and child problems (Tavassolie et al. 2016), thereby increasing parental stress. Therefore, it is reasonable to suggest that an individual's APP may be positively associated with the spouse's stress.

### 1.3 Moderating Role of Marital Satisfaction

Marital quality constitutes an important family context within which parents identify and fulfill their parental roles (Jiang et al. 2020). There are two contrasting propositions regarding how MS may affect the relationship between parenting perfectionism and parental stress. On the one hand, a stable and satisfactory marital relationship can provide parents with the strength and support necessary to fulfill parental responsibilities and manage parental stress (Hess 2008), even when they exhibit high levels of APP and/or MPP. Thus, an individual's perception of MS may buffer the positive associations between their MPP and both their own and their spouse's stress, as well as between mother‐reported APP and maternal stress, and may strengthen the negative association between father‐reported APP and paternal stress over time. In the context of high MS, one may also be more tolerant of the spouse's strict approach to parenting orderliness and more likely to recognize the spouse's positive intentions in socializing their children, thereby reducing their own parental stress. Hence, higher MS may weaken the positive associations between an individual's APP and the spouse's parental stress. On the other hand, couples in highly satisfactory marital relationships may be more emotionally attuned and responsive to each other's concerns (Hatfield et al. 1994). One parent may readily “catch” the spouse's perfectionistic stress and anxiety about parenting through emotional contagion (Hatfield et al. 1994), leading to a cycle of shared parental stress. According to this proposition, an individual's perception of MS may strengthen the positive associations between their MPP and both their own and their spouse's stress, as well as between their APP and their spouse's stress, and between mother‐reported APP and maternal stress. However, shared emotions between partners in the context of high MS may strengthen the protective function of father‐reported APP on paternal stress over time, as it fosters mutual understanding and social cohesion (Mazzuca et al. 2019).

### 1.4 The Current Study

The current study examined the patterns of father–mother interdependence in the associations of APP and MPP with parental stress among Chinese parents over time. Additionally, the moderating effects of father‐ and mother‐perceived MS in the associations were assessed. Two research questions were addressed:

Research Question 1: What are the patterns of father–mother interdependence in the associations of APP and MPP with paternal and maternal stress over time? Based on role identity theory (Cast 2004) and existing literature (Leung 2022), both father‐ and mother‐perceived MPP were positively related to their own parental stress. Furthermore, there is evidence of a crossover effect of mother‐perceived MPP on father's burnout (Brenning et al. 2024). Hence, we hypothesized that there would be an actor‐only pattern of father–mother interdependence in positive associations of MPP with maternal stress (H1a) and a couple pattern in positive associations of MPP with paternal stress (H1b) over time. Although empirical evidence shows that father‐perceived APP was negatively related to paternal stress, whereas mother‐perceived APP was positively related to maternal stress (Leung 2022), studies examining interspousal effects of APP on parental stress are virtually non‐existent; hence, analyses of these patterns are exploratory in nature.

Research Question 2: Does father‐ and mother‐perceived MS moderate the interdependent associations of APP and MPP with paternal and maternal stress over time? Given the contradictory perspectives between the “marital support” proposition (Hess 2008) and the “emotional contagion” thesis (Hatfield et al. 1994) regarding the moderating role of MS, these analyses are also exploratory in nature.

## 2 Method

### 2.1 Participants

A stratified cluster sampling method was used, secondary schools selected according to geographical location and school banding as stratifying factors. The inclusion criteria of respondents included: (a) intact families with children studying in Grades 7 and 8; (b) both fathers and mothers willing to participate in the study. Ultimately, eleven secondary schools participated. At Time 1 (T1), 642 Chinese couples with adolescent children were recruited. After one year (T2), the couples were invited to complete the questionnaire again, which was identical to that administered at T1. After matching, 506 sets of completed questionnaires were received from the couples, resulting in an attrition rate of 21.2%.

We conducted independent *t*‐tests to examine whether demographic variables (i.e., father's and mother's educational levels, number of children, family income, their children's gender and age) and the studied variables (i.e., father‐ and mother‐reported APP and MPP, paternal and maternal stress, and father‐ and mother‐perceived MS) at T1 differed between the dropout and retention samples. Results indicated a significant difference in adolescent age between the dropout and retention samples (*t* = 2.22, *p* < 0.05). However, no significant differences were found in any of the studied variables.

Among the 642 Chinese parent respondents at T1, the mean ages of fathers and mothers were 48.24 (SD = 6.80) and 44.02 (SD = 5.15), respectively. Of these, 37 (5.7%) fathers and 33 (5.3%) mothers had attained primary educational level or lower, 411 (64.0%) fathers and 424 (66.0%) mothers had reached secondary educational level, and 186 (28.9%) fathers and 180 (28.0%) mothers had achieved post‐secondary or university educational level (8 [1.2%] fathers and 4 [0.6%] mothers did not respond). Regarding the adolescent children, 371 (57.8%) were girls, with a mean age of 12.96 (SD = 0.98). In terms of family size, 147 (22.9%) couples had one child, 357 (55.6%) had two children, and 131 (20.4%) had three or more children (7 [1.1%] families did not respond). There were 178 (27.7%), 283 (44.2%), and 111 (17.3%) families having their monthly household income of less than HK$20,000 (US$2564), between HK$20,001 and HK$60,000 (US$2565–US$7692), and more than HK$60,001 (US$7693), respectively (17 [2.6%] families did not respond). Thirty‐six (5.6%) families were recipients of Comprehensive Social Security Assistance from the Hong Kong Government. The sample statistics closely resembled the demographic characteristics of Hong Kong population (Census and Statistics Department 2022).

### 2.2 Procedures

During data collection, we invited parents with adolescent children studying in Grades 7 and 8 to participate in the study. Written informed consent was obtained from each respondent. A package containing two questionnaires with a printed family code number and two envelopes was delivered to each couple via the participating schools. Respondents were asked to complete a self‐administered questionnaire comprising measures of APP, MPP, MS, parental stress, and demographic characteristics, and to place the completed questionnaire in a sealed envelope to ensure confidentiality. The participating schools collected the completed questionnaires and returned them to the researchers. Each couple received a HK$150 (US$42.5) supermarket coupon as a token of appreciation for their time and effort. After one year (T2), similar data collection procedures were conducted and the same questionnaire as at T1 was used. Ethical approval for the study was obtained from, and monitored by, the Human Subjects Ethics Sub‐committee of The Hong Kong Polytechnic University (Ref. No.: HSEARS20201012006).

### 2.3 Measurements

#### 2.3.1 Parenting Perfectionism

The Paternal and Maternal Self‐oriented Parenting Perfectionism Scale (PSPPS/MSPPS) was used. Drawing on the Multi‐dimensional Parenting Perfectionism Questionnaire developed by Snell Jr et al. (2005), Leung (2022) translated relevant items into Chinese to form the PSPPS and MSPPS, which were validated in a sample of Chinese parents, demonstrating good psychometric properties and measurement invariance across parent gender. Two higher‐order dimensions were identified: APP (orderliness in performing parenting duties) and MPP (exceptionally high parenting standards, over‐sensitivity of parenting mistakes, and doubts about one's parenting decisions; Leung 2022). Respondents rate each item on a 5‐point Likert scale from 1 = *strongly disagree* to 5 = *strongly agree*. A sample item for APP is “Orderliness is very important to me as a good parent.”, while a sample item for MPP is “If I make a parenting mistake, I will look down upon myself.” Both adaptive and maladaptive subscales of the PSPPS and the MSPPS at T1 showed good internal consistency in this study (Adaptive PSPPS: *α* = 0.80; Adaptive MSPPS *α* = 0.78; Maladaptive PSPPS: *α* = 0.87; Maladaptive MSPPS: *α* = 0.88).

#### 2.3.2 Parental Stress

Cheung (2000) developed a 17‐item Parental Stress Scale (PSS) to assess parental stress in performing parenting duties. The measure demonstrated good psychometric properties in a sample of Chinese parents in Hong Kong (Leung and Tsang 2010). Respondents rate the items on a 6‐point Likert scale from 1 = *strongly disagree* to 6 = *strongly agree*. A sample item reads “I feel overwhelmed by the responsibilities of being a parent.” Higher mean scores indicate higher levels of parental stress. Both the father‐ and mother‐reported PSS showed good internal consistency in this study (Father: *α* at T1 = 0.84; *α* at T2 = 0.87; Mother: *α* at T1 = 0.86, *α* at T2 = 0.89).

#### 2.3.3 Marital Satisfaction

The Chinese version of the Kansas Marital Satisfaction Scale (C‐KMS) was used based on the 3‐item Kansas Marital Satisfaction Scale (KMS) developed by Schumm et al. (1986), Shek and Tsang (1993) translated the measure into Chinese and validated it in a Chinese sample in Hong Kong, demonstrating good psychometric properties. Respondents rate each item on a 7‐point Likert scale of 1 = *very dissatisfied* and 7 = *very satisfied*. A sample item reads “How satisfied are you with your husband/wife as a spouse?” Higher mean scores indicate one's higher MS. The scale at T1 showed excellent reliability in this study (Father: *α* = 0.95; Mother: *α* = 0.96).

### 2.4 Data Analysis

**FIGURE 1:**
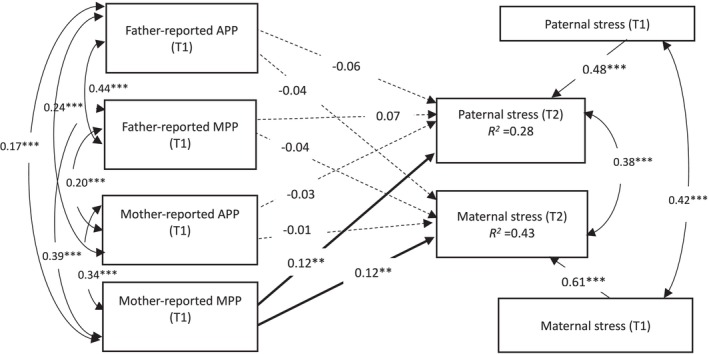
The actor–partner interdependence model in the associations of father‐ and mother‐reported parenting perfectionism with parental stress over time. APP, adaptive parenting perfectionism; MPP, maladaptive parenting perfectionism. Solid lines represent significant structural paths. Dotted lines represent non‐significant structural paths. Father's and mother's educational levels, gender and age of children, number of children and family income at T1 were controlled for. For simplicity, these covariates were not presented. **p < 0.01, ***p < 0.001.

To assess the patterns of father–mother interdependence in their associations of APP and MPP with parental stress over time, the steps suggested by Kenny and Ledermann (2010) were followed using AMOS 29.0. First, the saturated model was tested (see Figure 1). Goodness‐of‐fit indicators suggested by Hu and Bentler (1999) were adopted, that is, CFI > 0.90 and RMSEA < 0.06 for a good data fit. Next, we assessed the distinguishability of the dyad members by imposing equality constraints on the two actor effects and two partner effects of each dimension of parenting perfectionism on paternal and maternal stress, respectively. If a significant difference was observed between the constrained and saturated models, the dyadic members were considered distinguishable. We then calculated the ratio of partner to actor effect (*k*) by creating a phantom variable into each regression equation of the partner effect. By computing the confidence interval (CI) for each *k* value in 5000 bootstrapped resamples and determining whether 0, 1 or −1 was detected between 95% lower and upper CI, the patterns of associations (actor‐only, couple and contrast) of APP and MPP with paternal and maternal stress were determined. A partner‐only pattern was indicated as *k* ^−1^ = 0. We further constrained *k* to 1, 0 or −1 and tested the model again to verify the existence of the specific pattern. Finally, an equality constraint was set between *k*_father_ and *k*_mother_ to assess invariance between constrained and unconstrained models, if necessary. For indistinguishable members, the procedures suggested by Olsen and Kenny (2006) was adopted by creating an “interchangeable and saturated” model [ISAT; see Olsen and Kenny 2006 for details]. We computed the single *k* value and detected whether 0, 1 or −1 was found between 95% lower and upper CI. A partner‐only pattern was identified when *k* ^−1^ = 0.

**FIGURE 2:**
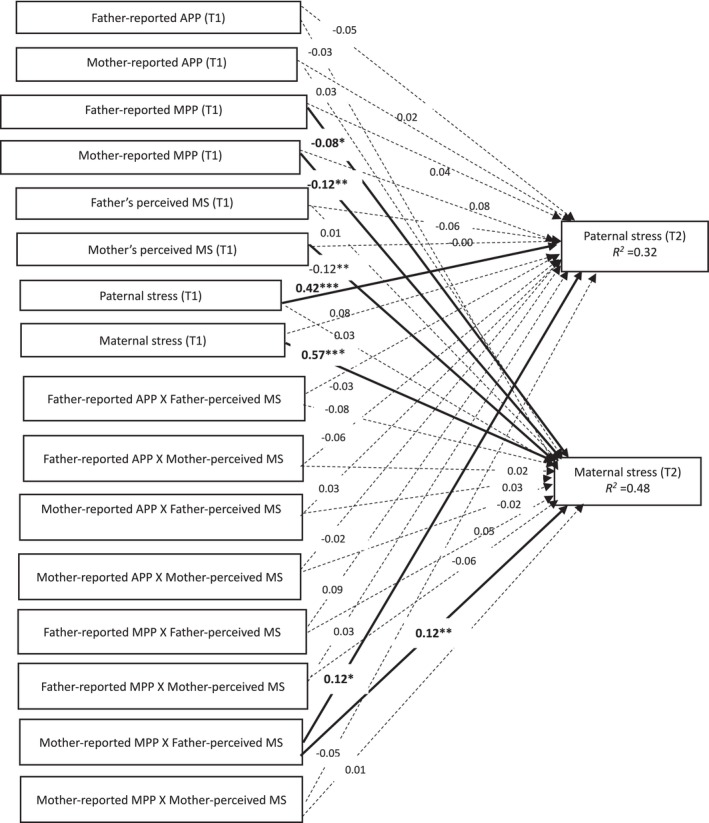
Moderation of father‐ and mother‐perceived marital satisfaction in the associations of father‐ and mother‐reported parenting perfectionism with parental stress over time. APP, adaptive parenting perfectionism; MPP, maladaptive parenting perfectionism; MS, marital satisfaction. Solid lines represent significant structural paths. Dotted lines represent non‐significant structural paths. Father's and mother's educational levels, gender and age of children, and family income at T1 were controlled for. For simplicity, these covariates were not presented. *p < 0.05, **p < 0.01, ***p < 0.001.

We adopted the APIM moderation model suggested by Garcia et al. (2015) to examine the moderating roles of father‐ and mother‐perceived MS in the interdependent relationships. Measures of father‐ and mother‐reported APP and MPP, and MS at T1 were mean‐centered respectively. Eight interaction terms were formulated (see Figure 2). When the interaction term significantly predicted either paternal or maternal stress at T2, the moderating effect was supported. Simple slope analyses and plotted graphs were used to illustrate the effects of father/mother‐perceived APP/MPP at T1 on paternal/maternal stress at T2 at high levels (1 SD higher than the mean) and low levels (1 SD lower than the mean) of father/mother‐perceived MS at T1.

## 3 Results

### 3.1 Preliminary Analyses

We conducted Little's Missing Completely at Random (MCAR) test (Little 1988) to determine whether the dataset was MCAR. The result was non‐significant [*χ* ^2^(28) = 38.08, *p* > 0.05], indicating that the dataset was indeed MCAR (Enders 2010). Given that the skewness and kurtosis values of all studied variables were below 2 and 7 respectively (Table 1), a normal distribution of the data was assumed (Curran et al. 1996). Therefore, Full Information Maximum Likelihood (FIML) estimation was applied to handle missing data.

**TABLE 1:** Descriptive statistics of the variables.

Variables	Range	Mean	SD	Skewness	Kurtosis
Father‐reported APP (T1)	1–5	3.54	0.68	−0.74	1.04
Mother‐reported APP (T1)	1–5	3.59	0.64	−0.72	0.67
Father‐reported MPP (T1)	1–5	2.45	0.58	0.25	0.47
Mother‐reported MPP (T1)	1–5	2.51	0.61	0.37	0.34
Father‐perceived MS (T1)	1–7	5.50	1.06	−0.58	0.38
Mother‐perceived MS (T1)	1–7	5.09	1.15	−0.68	0.89
Paternal stress (T1)	1–6	2.70	0.57	0.11	−0.29
Maternal stress (T1)	1–6	2.74	0.62	0.19	−0.26
Paternal stress (T2)	1–6	2.78	0.59	−0.19	−0.36
Maternal stress (T2)	1–6	2.77	0.67	0.16	−0.31
Child gender (T1) (1 = boys, 2 = girls)	1–2	1.58	0.49	−0.32	−1.91
Child age (T1)	11–19	12.96	0.98	1.10	2.73
Father's educational level (T1)	1–6	4.08	1.19	0.26	−0.68
Mother's educational level (T1)	1–6	4.08	1.08	0.20	−0.32
Family income (T1)	1–7	3.93	1.92	0.39	−1.15
Number of children (T1)	1–5	2.02	0.77	0.79	1.28

Abbreviations: APP, adaptive parenting perfectionism; MPP, maladaptive parenting perfectionism; MS, marital satisfaction.

Correlational analyses revealed that father‐reported APP at T1 was negatively and significantly associated with both paternal and maternal stress at T1, but not at T2. Mother‐reported APP at T1 was not associated with paternal or maternal stress at either time point. Both father‐reported and mother‐reported MPP at T1 were positively correlated with both paternal and maternal stress at both time points (Table 2).

**TABLE 2:** Correlations of the variables.

	1	2	3	4	5	6	7	8	9	10	11	12	13	14	15
1. Father‐reported APP (T1)															
2. Mother‐reported APP (T1)	0.24***														
3. Father‐reported MPP (T1)	0.41***	0.20***													
4. Mother‐reported MPP (T1)	0.13***	0.34***	0.39***												
5. Father‐perceived MS (T1)	0.19***	0.12**	−0.07	−0.04											
6. Mother‐perceived MS (T1)	0.12**	0.06	−0.10**	−0.13**	0.54***										
7. Paternal stress (T1)	−0.10*	−0.00	0.31***	0.24***	−0.37***	−0.25***									
8. Maternal stress (T1)	−0.08*	0.02	0.17***	0.42***	−0.17***	−0.37***	0.42***								
9. Paternal stress (T2)	−0.07	0.02	0.24***	0.24***	−0.24***	−0.18***	0.52***	0.31***							
10. Maternal stress (T2)	−0.08	0.00	0.11*	0.34***	−0.16***	−0.35***	0.28***	0.66***	0.45***						
11. Child gender (T1)	−0.02	0.07	0.03	0.06	−0.00	0.01	−0.04	−0.01	−0.02	0.02					
12. Child age (T1)	0.06	−0.01	0.06	0.06	−0.06	−0.03	0.05	0.01	0.10*	0.03	−0.02				
13. Father's educational level (T1)	0.09*	0.04	−0.01	−0.08	0.05	0.06	−0.06	0.00	−0.11*	−0.02	−0.04	−0.10*			
14. Mother's educational level (T1)	0.02	0.04	−0.11**	−0.12**	0.08*	0.07	−0.06	−0.05	−0.08	−0.03	−0.11**	−0.16***	0.53***		
15. Family income (T1)	0.01	0.02	−0.16***	−0.18***	0.07	0.15***	−0.08	−0.08*	−0.08	−0.08	−0.04	−0.16***	0.40***	0.48***	
16. Number of children (T1)	−0.01	−0.04	0.06	−0.02	0.02	0.05	0.01	0.04	0.04	0.08	0.05	0.03	−0.08*	−0.04	−0.06

Abbreviations: APP, adaptive parenting perfectionism; MPP, maladaptive parenting perfectionism; MS, marital satisfaction.

* *p* < 0.05.

** *p* < 0.01.

*** *p* < 0.001.

### 3.2 Patterns of Father–Mother Interdependence in Associations of Parenting Perfectionism With Parental Stress Over Time

Regarding father‐ and mother‐reported MPP as predictors, the tested model (M1) showed a good data fit, with *x* ^2^(39) = 52.005 (*p* > 0.05), CFI = 0.992, RMSEA = 0.023 [90% CI (0.000, 0.038)]. After constraining the two actor effects and two partner effects to be equal (M2), M2 and M1 differed significantly [Δ*x* ^2^(2) = 6.581, *p* < 0.05], indicating that dyadic members were distinguishable (Kenny and Ledermann 2010). Mother‐reported MPP was positively related to maternal stress (*β* = 0.12, *p* < 0.01) and paternal stress (*β* = 0.12, *p* < 0.01) over time, whereas father‐reported MPP was neither associated with paternal stress (*β* = 0.07, *p* > 0.05) nor maternal stress (*β* = −0.04, *p* > 0.05; Figure 1).

For father‐ and mother‐reported APP as predictors, after constraining the two actor effects and two partner effects to be equal (M3), no significant difference was found between M3 and M1 [Δ*x* ^2^(2) = 0.738, *p* > 0.05], indicating indistinguishable dyads (Kenny and Ledermann 2010). However, all four regression paths were non‐significant (Figure 1). The regression paths are trivial (*β* < 0.10; Kenny and Ledermann 2010) for examining patterns of father–mother interdependence in the associations of APP with paternal and maternal stress over time.

To examine the pattern of interdependence between MPP and parental stress, we created a phantom variable between father‐reported MPP and maternal stress (*k*_Mother_), and another phantom variable (actor to partner ratio, $k_{\text{Father}}^{-1}$) between father‐reported MPP and paternal stress (i.e., the actor effect; Kenny and Ledermann 2010) in the tested model (M4). M4 showed a good data fit, with *x* ^2^(39) = 48.703 (*p* > 0.05), CFI = 0.994 and RMSEA = 0.020 [90% CI (0.000, 0.036)]. The standardized regression coefficient of *k*_Mother_ was −0.018, with a 95% CI ranging from −0.090 to 0.054, including zero within the interval. When we fixed *k*_Mother_ at “0” (M5), the difference between M5 and M4 was non‐significant [Δ*x* ^2^(1) = 0.218, *p* > 0.05], suggesting an actor‐only pattern in the relationship between MPP and maternal stress. For paternal stress, the standardized regression coefficient of $k_{\text{Father}}^{-1}$ was 0.079, with a 95% CI ranging from −0.001 to 0.159 (including zero within the interval). After fixing $k_{\text{Father}}^{-1}$ to zero (M6), the difference between M6 and M4 was non‐significant [Δ*x* ^2^(1) = 3.268, *p* > 0.05], indicating a partner‐only pattern in the relationship between MPP and paternal stress. In summary, the positive association of MPP with paternal stress demonstrated a partner‐only pattern, while the positive association of MPP with maternal stress was an actor‐only pattern over time. H1a was supported and H1b was not supported.

### 3.3 Moderating Roles of Father‐ and Mother‐Perceived Marital Satisfaction (MS)

**FIGURE 3:**
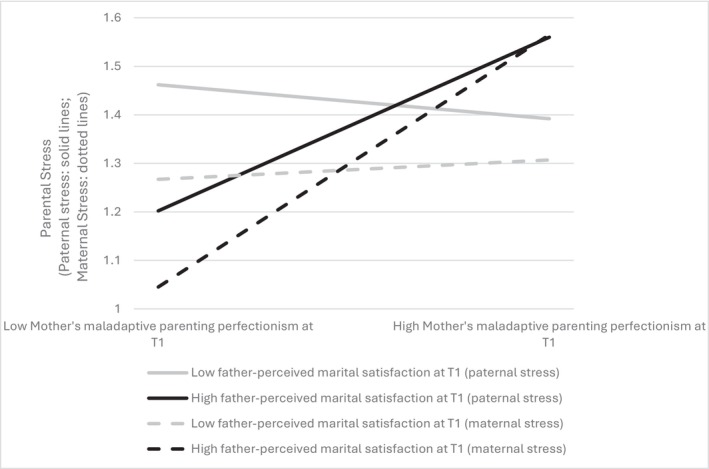
Regression of parental stress by mother‐reported maladaptive parenting perfectionism in high and low levels of father‐perceived marital satisfaction.

The tested model (M7; Figure 2) showed a good data fit, with *x* ^2^ (146) = 288.115, *p* < 0.001, NFI = 0.934, CFI = 0.965 (> 0.90; Hu and Bentler 1999) and RMSEA = 0.039 (< 0.06; Hu and Bentler 1999). Results indicated that the interaction term “mother‐reported MPP X father‐perceived MS” was positively associated with both paternal and maternal stress at T2, with *β*s = 0.12 (*p* < 0.05) and 0.12 (*p* < 0.05) respectively. At higher levels of father‐perceived MS, the associations of mother‐reported MPP with paternal and maternal stress were *β*s = 0.20 (*p* < 0.01) and 0.25 (*p* < 0.001) respectively. Conversely, at lower levels of father‐perceived MS, the associations of mother‐reported MPP with paternal and maternal stress were not significant, with *β*s = −0.05 (*p* > 0.05) and 0.01 (*p* > 0.05), respectively. Figure 3 illustrates the associations of mother‐reported MPP with paternal and maternal stress at high (1 SD higher than the mean) and low (1 SD lower than the mean) levels of father‐perceived MS. In summary, father‐perceived MS strengthened the positive associations of mother‐reported MPP with paternal and maternal stress over time.

## 4 Discussion

The study aimed to examine the patterns of father–mother interdependence in the relationship between parenting perfectionism and parental stress over time. Moreover, the moderating roles of father‐ and mother‐perceived MS in the associations were assessed. The findings revealed a partner‐only pattern in the positive association of MPP with paternal stress, and an actor‐only pattern in the positive association of MPP with maternal stress over time.

The findings provide empirical support for role identity theory (Cast 2004), suggesting that mothers who exhibit MPP may feel inadequate and doubtful about their parenting decisions, and may be fearful of making mistakes, which can impede their role identity as “ideal parents” and thus generate parental stress (Scarnier et al. 2009). As mothers are typically main caregivers within the family, they may be more inclined towards MPP when they fear that any parenting imperfection could jeopardize their children's future (Liss et al. 2013). Furthermore, mother‐reported MPP was also associated with paternal stress over time, echoing the findings of Brenning et al. (2024) that fathers may experience stress and fatigue when confronted with extremely high standards set by their spouse. However, in contrast to mother‐reported MPP, father‐reported MPP was not related to either their own and their spouse's parental stress. As mentioned, fathers tend to be more goal‐directed and instrumental in parenting (Power and Shanks 1989). When fathers exhibit high levels of MPP, they may modify their parenting strategy to fulfill their parental goals, thereby managing their stress more effectively (Leung 2022).

It is noteworthy that, in contrast to mother‐reported MPP, neither father‐ nor mother‐reported APP predicted paternal or maternal stress over time. Although parenting orderliness may enhance parents' sense of self‐control and self‐efficacy (Lee et al. 2012), excessive rigidity in parenting orderliness may also result in inflexibility and parent–child conflict (Everri et al. 2016), particularly when adolescent children seek greater independence, which may create parental stress. A cancel‐out effect may therefore occur. Indeed, previous research has identified only a weak, albeit significant, relationship between APP and parental stress (Leung 2022).

The findings further indicated that father‐perceived MS strengthened the positive associations of mother‐reported MPP with paternal and maternal stress over time, supporting the proposition of emotional contagion (Hatfield et al. 1994). Fathers perceiving higher MS are more likely to “catch” their spouse's perfectionistic anxiety about parenting and to synchronize their spouse's emotions, resulting in increased paternal stress. Paternal stress may feed back to mothers, which creates maternal stress via emotional contagion (Hatfield et al. 1994), forming a cycle of shared perfectionistic anxiety.

The findings also suggest that the process of emotional contagion within the family system may be specific to certain forms of parenting perfectionism and conditions of MS. Mother‐reported MPP appears to be a particularly potent source of paternal and maternal stress, especially when fathers perceive their marital relationship positively. In contrast, father‐reported APP and MPP, and mother‐reported APP do not activate paternal and maternal stress over time, suggesting that these forms of parental perfectionism are less likely to trigger emotional contagion, even in highly satisfied marital contexts. Fathers tend to adopt a more instrumental role in parenting (Power and Shanks 1989) and appear to be more reserved in sharing their parenting anxieties with their spouse (Louie 2002), which may result in mothers not synchronizing with paternal stress. Furthermore, fathers may not even recognize maternal stress arising from APP, as its magnitude may be too small to detect. Given the limited research on parenting perfectionism, MS, parental stress and emotional contagion, further studies in this area are recommended.

There are several limitations in the study. Firstly, it focused primarily on self‐oriented parenting perfectionism as a predictor of parental stress, neglecting socially prescribed parenting perfectionism (i.e., high social expectations to be an ideal parent; Snell Jr et al. 2005). There is a need to examine different facets of parenting perfectionism in relation to parental stress. Secondly, the study did not explore mediating mechanisms (e.g., parental guilt, shame, and anxiety) through which MPP affects parental stress. Thirdly, data were collected solely through self‐reported questionnaire, which may introduce shared method variance and inflate the observed relationships. Qualitative methods (e.g., case interview) could be employed to corroborate these findings. Fourthly, attrition analyses have indicated a significant difference in adolescent age between dropout and retention samples, which may introduce bias. Lastly, the study was conducted in Hong Kong. Replicating the study in other Chinese communities (e.g., the Chinese mainland, Taiwan, American Chinese) and other Asian countries (e.g., Japan, South Korea) is necessary to enhance the generalisability of the findings.

Despite these limitations, the study makes several theoretical and practical contributions. The rise of global meritocracy and rapid social changes have made parenting in the millennium increasingly challenging (Leung 2022). Indeed, studies examining parenting perfectionism and parents' well‐being remain limited. This study is novel in its examination of patterns of father–mother interdependence in the associations between parenting perfectionism and parental stress among Chinese families over time. Moreover, the study assesses the moderating role of father‐ and mother‐perceived MS in the interdependent associations, thereby providing important evidence for the literature. The findings support the social contagion proposition (Hatfield et al. 1994) that father‐perceived MS strengthens the positive associations of mother‐reported MPP with both paternal and maternal stress over time, thereby contributing to the development of Chinese models of family dynamics and well‐being.

Practically, the findings revealed an actor‐only pattern of MPP on maternal stress and a partner‐only pattern on paternal stress, suggesting that mother‐perceived MPP is a risk factor for increased stress within Chinese families. Family therapists and social workers should support mothers in alleviating perfectionistic anxiety about parenting through parental education and counseling. Moreover, as father‐perceived MS may strengthen these associations, involving fathers in family counseling to facilitate mutual understanding and clear boundary‐setting is recommended.

## Funding

This research was financially supported by the General Research Fund, Research Grants Council (Project Code: PolyU 15605021) and Internal Research Fund of The Hong Kong Polytechnic University (No.: 1‐W01V).

## Conflicts of Interest

The authors declare no conflicts of interest.

## Data Availability

Datasets generated for this research are available upon request to the corresponding author on reasonable request.
